# Prognostic indicators of disease progression in Duchenne muscular dystrophy: A literature review and evidence synthesis

**DOI:** 10.1371/journal.pone.0265879

**Published:** 2022-03-25

**Authors:** Nermina Ferizovic, Jessica Summers, Igor Beitia Ortiz de Zárate, Christian Werner, Joel Jiang, Erik Landfeldt, Katharina Buesch

**Affiliations:** 1 MAP BioPharma Ltd, Cambridge, England, United Kingdom; 2 BresMed Health Solutions, Sheffield, England, United Kingdom; 3 PTC Therapeutics France SAS, Paris, France; 4 PTC Therapeutics Germany GmbH, Frankfurt/Main, Germany; 5 PTC Therapeutics, South Plainfield, New Jersey, United States of America; 6 ICON plc, Stockholm, Sweden; 7 PTC Therapeutics Switzerland GmbH, Zug, Switzerland; Faculty of Medical Science - State University of Campinas, BRAZIL

## Abstract

**Background:**

Duchenne muscular dystrophy (DMD) is a rare, severely debilitating, and fatal neuromuscular disease characterized by progressive muscle degeneration. Like in many orphan diseases, randomized controlled trials are uncommon in DMD, resulting in the need to indirectly compare treatment effects, for example by pooling individual patient-level data from multiple sources. However, to derive reliable estimates, it is necessary to ensure that the samples considered are comparable with respect to factors significantly affecting the clinical progression of the disease. To help inform such analyses, the objective of this study was to review and synthesise published evidence of prognostic indicators of disease progression in DMD. We searched MEDLINE (via Ovid), Embase (via Ovid) and the Cochrane Library (via Wiley) for records published from inception up until April 23 2021, reporting evidence of prognostic indicators of disease progression in DMD. Risk of bias was established with the grading system of the Centre for Evidence-Based Medicine (CEBM).

**Results:**

Our search included 135 studies involving 25,610 patients from 18 countries across six continents (Africa, Asia, Australia, Europe, North America and South America). We identified a total of 23 prognostic indicators of disease progression in DMD, namely age at diagnosis, age at onset of symptoms, ataluren treatment, ATL1102, BMI, cardiac medication, DMD genetic modifiers, DMD mutation type, drisapersen, edasalonexent, eteplirsen, glucocorticoid exposure, height, idebenone, lower limb surgery, orthoses, oxandrolone, spinal surgery, TAS-205, vamorolone, vitlolarsen, ventilation support, and weight. Of these, cardiac medication, DMD genetic modifiers, DMD mutation type, and glucocorticoid exposure were designated core prognostic indicators, each supported by a high level of evidence and significantly affecting a wide range of clinical outcomes.

**Conclusion:**

This study provides a current summary of prognostic indicators of disease progression in DMD, which will help inform the design of comparative analyses and future data collection initiatives in this patient population.

## 1. Introduction

Duchenne muscular dystrophy (DMD) is a rare, neuromuscular disease characterised by progressive muscle degeneration caused by mutations in the X-linked *DMD* gene [1, 2]. The *DMD* gene encodes dystrophin, a structural protein which forms part of complexes predominantly found in muscle cells where it plays a significant role in the stabilisation of cell membranes [3]. To date, over 1,100 mutations have been identified, including 891 responsible for DMD phenotypes [4]. The incidence of DMD has been estimated at between 1 in 3,500 and 5,000 live male births [5, 6].

Patients with DMD are diagnosed around the age of four years, but many boys show symptoms earlier due to proximal muscle weakness resulting in delayed physical milestones (e.g., walking, running, and climbing stairs). As the disease progresses, patients become non-ambulatory usually in their early teens, followed by increasing loss of upper limb strength and function [7–11]. Respiratory and cardiac decline ensue, with patients eventually requiring mechanical ventilation support for survival [9, 10]. The median life expectancy at birth is around 30 years [12]. At present, there is no cure for DMD, and standard of care is mainly aimed at managing disease symptoms and promoting patient quality of life [13].

In medical research, it is occasionally necessary to pool patient-level data from different studies to indirectly assess the efficacy of a treatment due to low statistical power because of small patient samples and/or the absence of direct comparators in randomised controlled trials (RCTs). To minimize bias in such analyses, it is important to ensure that the populations to be compared are sufficiently homogeneous with respect to factors that would be expected to directly or indirectly affect outcomes of interest [14]. For example, in the context of DMD, it would be relevant to adjust any indirect comparison for the current age of the patient, among other factors, given the progressive, age-related nature of the disease. However, to date, no study has systematically reviewed the body of evidence for factors affecting disease progression outcomes in DMD. To bridge this evidence gap, the objective of this study was to review and synthesise the published evidence on prognostic indicators of disease progression in DMD.

## 2. Methods

This literature review was conducted and reported in accordance with the Preferred Reporting Items for Systematic Reviews and Meta-Analyses (PRISMA) statement [15]. The study protocol is not publicly available due to intellectual property restrictions.

### 2.1. Search strategy

We searched MEDLINE (via Ovid), Embase (via Ovid) and the Cochrane Library (via the Wiley online platform) for records of studies published from inception up until April 23 2021, reporting evidence of prognostic indicators of disease progression in DMD. The search string contained “Duchenne muscular dystrophy” as a Medical Subject Heading term or free text term in combination with variations of the free text term “prognostic indicator”. For example, the MEDLINE population terms were: 1. “exp Muscular Dystrophy, Duchenne/”, 2. “(Duchenne and dystro*).mp.” and 3. “1 or 2”. These were combined with the prognostic indicator terms; 4. “(prognos* or (disease adj3 course) or (disease adj3 impact) or natural history or (disease adj3 predict*) or (disease adj3 outcome) or (disease adj3 progres*)).mp.” and 5. “3 and 4”. Then the searches filtered out irrelevant study designs with the following; 6. “(comment or letter or editorial or notes or review).pt.”, 7. “(exp animals/ or exp invertebrate/ or animal experiment/ or animal model/) and (human/)” and 8. “(exp animals/ or exp invertebrate/ or animal experiment/ or animal model/) not 7”, 9. “6 or 8” and 10. “5 not 9”. Full search strings are provided in S1 Appendix.

### 2.2. Selection criteria

Eligibility criteria based on the Population, Intervention, Comparison, Outcomes and Study design (PICOS) framework for study inclusion are presented in Table 1. Only English language texts were included. For the purposes of this review, a prognostic indicator was defined as any factor, either endogenous or exogenous, affecting the clinical progression of disease.

**Table 1:** PICOS eligibility criteria for study inclusion.

	Inclusion	Exclusion
**Population**	Patients diagnosed with DMD	Patients without a diagnosis of DMD
**Intervention**	Any	None
**Comparators**	Any	None
**Outcome**	Prognostic indicator of disease progression	None
**Study design**	Any	Systematic literature reviews and meta-analyses were not formally included, but screened for relevant references

Note: Population, Intervention, Comparison, Outcomes and Study design (PICOS). Duchenne muscular dystrophy (DMD).

### 2.3. Screening and data extraction

One investigator (NF) initially screened article titles and abstracts for eligibility, and subsequently reviewed full-text versions of selected records. The reason for exclusion was recorded and confirmed by a second investigator (JS). For all articles that met the inclusion criteria upon full-text review, the following information was extracted into a pre-designed data extraction form: Author, year, geographical setting, study design, interventions, patient sample population characteristics, disease progression outcome measures, prognostic indicators, and the impact of the prognostic indicators on disease progression. For the purpose of this review, we only considered statistically significant prognostic indicators (as reported in the included studies).

We synthesised extracted evidence of the impact of identified prognostic indicators of disease progression in DMD into eight outcome categories: cardiac health and function, loss of independent ambulation, lower extremity and motor function, muscle strength, respiratory health and function, scoliosis, survival, and upper extremity function. Although loss of ambulation is a clinical milestone within the lower extremity and motor function domain, we decided to report evidence separately for this factor given its central role in DMD research (e.g., as a primary endpoint in RCTs). Due to the monotonic progression of DMD, we did not consider current age a prognostic factor of interest, nor bisphosphonate therapy because of the negative impact from both glucocorticoids and DMD on bone health [13].

### 2.4. Level of evidence

The level of evidence of included studies was established using a modified version of the grading system of the Centre for Evidence-Based Medicine (CEBM) [16]. Specifically, five levels of evidence were designated based on study design: (1) systematic review of randomised trials or n-of-1 trials, (2) randomised trial or observational study with dramatic effect, (3) non-randomised controlled cohort/follow-up study, (4) case-series, case-control studies, or historically controlled studies, and (5) mechanism-based reasoning. For reporting purposes, we categorised evidence levels 1 and 2 as “high level of evidence”, level 3 as “moderate level of evidence”, and levels 4 and 5 as “low level of evidence”.

## 3. Results

**Fig 1:**
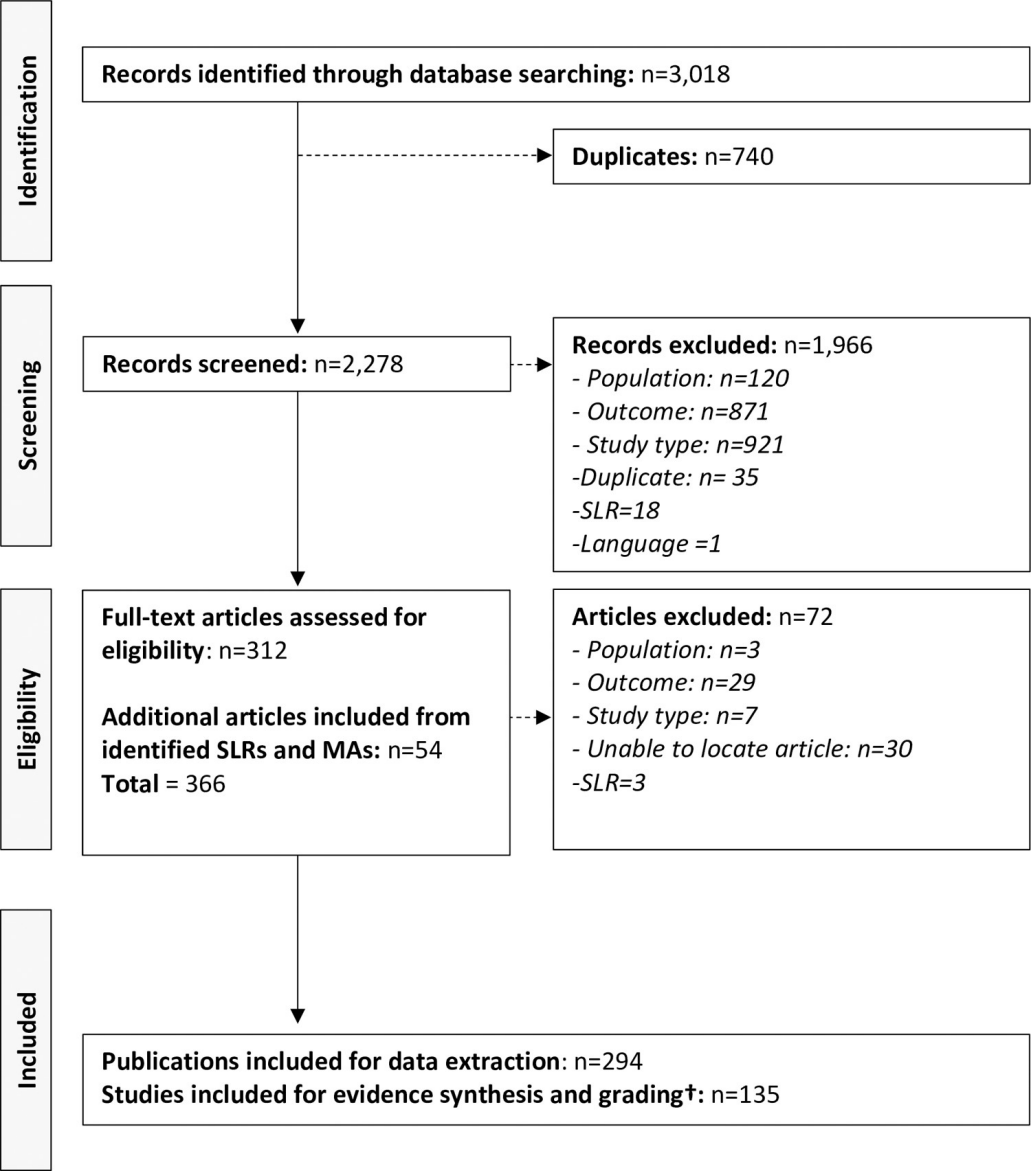
PRISMA diagram of the selection process of the included publications. Note: † Studies reporting evidence of statistically significant prognostic indicator of disease progression in DMD. Systematic literature reviews (SLRs). Meta-analyses (MAs).

The search was performed on April 26 2021, and resulted in the identification of 3,018 publications (including journal articles and congress/conference abstracts) reporting evidence of prognostic indicators of disease progression in DMD (Fig 1). Of these, 740 records were duplicates, 1,966 excluded following title and abstract screening, and 312 selected for full-text review. An additional 54 articles were included from the reference searches of identified systematic literature reviews (SLRs) and meta-analyses (MAs). Finally, 294 publications were considered for data extraction, with 135 studies reporting statistically significant prognostic indicators of disease progression that were subsequently included for evidence synthesis and grading. Summary details of the included studies are presented in Table 2. Identified studies encompassed 25,610 patients with DMD from 18 countries (Argentina, Australia, Belgium, Canada, China, Denmark, Egypt, France, Germany, Holland, India, Italy, Japan, Korea, Sweden, Turkey, the United Kingdom and the United States).

**Table 2:** Characteristics of included studies and identified prognostic indicators in DMD.

Author, year (country)	Study design (level of evidence)†	Interventions, DMD genetic modifiers, and/or DMD mutation types	Patient population	Disease progression outcome category	Disease progression outcome results	Identified prognostic indicator‡
Biggar et al., 2006 (CA) [17]	Non-randomised controlled cohort(Level 3)	DFZ	74 patients with DMD (mean age: NR, range: 10–18 years)	Cardiac Health and Function	Improved fractional shortening and ejection fraction	Glucocorticoid exposure
				Respiratory Health and Function	Improved and sustained FVC	
Houde et al., 2008 (CA) [18]	Case-control study (Level 4)	DFZ	79 patients with DMD treated with DFZ (mean age: 13 years, range: NR) or no treatment (mean age: 18 and 10 years, range: NR)	Cardiac Health and Function	Improved fractional shortening, ejection fraction, and reduced risk of cardiomyopathy	Glucocorticoid exposure
				Scoliosis	Lower mean degrees of scoliosis	
				Loss of Ambulation	Delay in loss of ambulation	
				Respiratory Health and Function	Improved FVC	
				Muscle Strength	Improved muscle strength as given by MRC	
Silversides et al., 2003 (CA) [19]	Case-control study (Level 4)	DFZ	33 patents with DMD treated with DFZ (mean age: 14 years, range: 10–18 years) or no treatment (mean age: 16 years, range: 11–18 years)	Cardiac Health and Function	Improved fractional shortening, ejection fraction, and LVEDd	Glucocorticoid exposure
				Respiratory Health and Function	Preserved pulmonary function	
				Loss of Ambulation	Delay in loss of ambulation	
Barber et al., 2013 (US) [20]	Case-control study (Level 4)	DFZ and PDN/PRED	462 patients with DMD (mean age: NR, range: NR)	Cardiac Health and Function	Reduced risk of cardiomyopathy onset versus untreated and linked to duration of use	Glucocorticoid exposure
				Loss of Ambulation	Delay in loss of ambulation linked to duration of use	
Bello et al., 2019 (IT) [21] Bello et al., 2019 (IT) [22]	Case series (Level 4)	DFZ and PDN/PRED LTBP4, minor alleles at SPP1, and CD40 SNPs Dp140 and Exon 8 skipping	374 patients with DMD (mean age: NR, range: NR)	Cardiac Health and Function	*DFZ and PDN/PRED* Improved ejection fraction *LTBP4* Preserved ejection fraction	Glucocorticoid exposure; DMD genetic modifiers; and DMD mutation type
				Respiratory Health and Function	*Dp140* Reduced FVC *DFZ and PDN/PRED* Improved FVC *SPP1 and CD40 SNPs* Reduced FVC *Exon 8 skipping* Higher PEF	
Tandon et al., 2015 (US) [23]	Case series (Level 4)	DFZ and PDN/PRED	98 patients with DMD (mean age: NR, range: NR)	Cardiac Health and Function	Decline in LVEF linked to duration of use	Glucocorticoid exposure
Zhang et al., 2015 (CN) [24]	Non-randomised controlled cohort study (Level 3)	DFZ and PDN/PRED	77 patients with DMD (mean age: NR, range: 2–13 years)	Cardiac Health and Function	Increased summed rest score	Glucocorticoid exposure
Schram et al., 2013 (CA) [25]	Case series (Level 4)	DFZ and PDN/PRED All patients were receiving cardiac medication (ACE inhibitors/ARBs)	86 patients with DMD (mean age: NR, range: NR)	Cardiac Health and Function	Reduced risk of cardiomyopathy, improved fractional shortening, ejection fraction, and LVEDd	Glucocorticoid exposure
				Survival	Reduction in all-cause mortality	
Markham et al., 2008 (US) [26]	Case-control study (Level 4)	DFZ and PDN/PRED	37 patients with DMD (mean age: NR, range: NR)	Cardiac Health and Function	Improved LVEDd, shortening fraction, mWS, and VCFc	Glucocorticoid exposure
Markham et al., 2005 (US) [27]	Case-control study (Level 4)	DFZ and PDN/PRED	111 patients with DMD treated with DFZ and PDN/PRED (mean age: 11 years, range: 3–21 years) or no treatment (mean age: 12 years, range: 3–21 years)	Cardiac Health and Function	Improved fractional shortening	Glucocorticoid exposure
Kim et al., 2017 (US) [28]	Case series (Level 4)	DFZ and PDN/PRED	255–660 patients with DMD (mean age: NR, range: NR)	Cardiac Health and Function	Increased risk of cardiomyopathy linked to duration of use	Glucocorticoid exposure
				Respiratory Health and Function	Reduced FVC function linked to duration of use	
Aikawa et al., 2019 (JP) [29]	Case series (Level 4)	ACE inhibitor (cilazapril or enalapril)	21 patients with DMD (median age: 12 years, IQR: 6–16 years)	Cardiac Health and Function	Improved LVEF	Cardiac medication
Kwon et al., 2012 (KR) [30]	Randomised trial (Level 2)	ACE inhibitor (enalapril) or BB (carvedilol)	23 patients with DMD (mean age: 13 years, range: NR)	Cardiac Health and Function	*BB* Improved LVMPI *ACE* Improved LVESd and left ventricular free wall systolic myocardial velocity	Cardiac medication
Kajimoto et al., 2006 (JP) [31]	Non-randomised controlled cohort (Level 3)	ACE inhibitor (enalapril), or ACE inhibitor (enalapril) and BB (carvedilol)	25 patients with DMD treated with ACE inhibitors/BBs (mean age: 18 years, range: 7–27 years) or ACE inhibitors (mean age: 15 years, range 8–29 years)	Cardiac Health and Function	*ACE* Improved LVEDd *ACE/BB* Improved LVFS	Cardiac medication
Thrush et al., 2012 (US) [32] Thrush et al., 2012 (US) [33]	Case-control study (Level 4)	ACE inhibitor (drug NR), or ACE inhibitor (drug NR) and BB (drug NR)	25 patients with DMD treated with ACE inhibitors/BBs (mean age: 16 years, range: NR) or ACE inhibitors (mean age: 14 years, range: NR)	Cardiac Health and Function	Both ACE inhibitor and ACE inhibitor/BB improved ejection fraction compared to natural history	Cardiac medication
Viollet et al., 2012 (US) [34]	Case-control study (Level 4)	ACE inhibitor (lisinopril), or ACE inhibitor (lisinopril) and BB (metoprolol)	54 patients with DMD treated with ACE inhibitors/BBs (mean age: 16 years, range: 10–24 years) or ACE inhibitors (mean age: 14 years, range: 7–27 years)	Cardiac Health and Function	Improved ejection fraction versus natural history control	Cardiac medication
Jefferies et al., 2005 (US) [35]	Case series (Level 4)	ACE inhibitor (drug NR) and BB (drug NR)	62 patients with DMD (mean age: NR, range: NR)	Cardiac Health and Function	Improved LVEDd, LVEF, LVMPI, and left ventricular sphericity index	Cardiac medication; and DMD mutation type
		Exon 51 and 52			Cardioprotective	
		Exon 12,14, 15, 16, and 17			Onset of cardiomyopathy	
Raman et al., 2015 (US) [36]	Randomised trial (Level 2)	EPL and PLC	42 patients with DMD treated with EPL (mean age: 15 years, range: 12–19 years) or PLC (mean age: 15 years, range: 11–19 years)	Cardiac Health and Function	Improved left ventricular systolic strain, LVEF, and ESV	Cardiac medication
Matsumura et al., 2010 (JP) [37]	Non-randomised controlled cohort study (Level 3)	BB	54 patients with DMD treated with BBs (mean age: 19 years, range: 11–29 years) or BSC (mean age: 23 years, range: 15–35 years)	Cardiac Health and Function	Reduction in heart failure and arrhythmias	Cardiac medication
Van Ruiten et al., 2017 (UK) [38]	Case control (Level 4)	Cardiac medication (drug NR)	108 patients with DMD (mean age: NR, range: NR)	Cardiac Health and Function	Timing of cardiac medication impacts on cardiomyopathy	Cardiac medication
		DFZ and PDN/PRED		Loss of Ambulation	Delay in loss of ambulation	Glucocorticoid exposure
				Respiratory Health and Function	Improved FVC	
Fayssoil et al., 2018 (FR) [39]	Case series (Level 4)	Ventilation support in combination with cardiac medication (drug NR)	101 patients with DMD (median age: 21 years, IQR: 18–26 years)	Cardiac Health and Function	Decreased left atrium diameter and LVEF	Ventilation support
Nagai et al., 2020 (JP) [40]	Case-control study (Level 4)	ACTN3 null genotype	77 patients with DMD (median age: NR; IQR: 7.9–11.5 years)	Cardiac Health and Function	Earlier onset of cardiac dysfunction; early onset of LV dilation; lower LV dilation-free rate	DMD genetic modifier
Cheeran et al., 2017 (US) [41]	Case-control study (Level 4)	BMI	43 patients with DMD (median age: 21 years; IQR: 21–24 years)	Cardiac Health and Function	Higher BMI is associated with reduced cardiomyopathy	BMI
Duboc et al., 2005 (FR) [42] Duboc et al., 2007 (FR) [43]	Randomised trial (Level 2)	Perindopril and PLC	57 patients with DMD (mean: NR; range: 9.5–13 years)	Cardiac Health and Function	Maintains LVEF	Cardiac medication
				Survival	Improvement in survival	
Ishikawa et al., 1999 (NR) [44]	Follow-up study (Level 3)	ACE (enalapril and lisinopril) and BB	11 patients with DMD (mean age: 17; range: 12.6–22.8)	Cardiac Health and Function	Increased LVEF	Cardiac medication
Ramaciotti et al., 2006 (USA) [45]	Case-series (Level 4)	ACE (enalapril)	50 patients with DMD (mean age: NR; range: 10–20 years)	Cardiac Health and Function	Improved left ventricular function	Cardiac medication
King et al., 2007 (US) [46]	Case-control study (Level 4)	DFZ and PDN/PRED	143 patients with DMD treated with DFZ and PDN/PRED (mean age: 17 years, range: 6–31 years) or no treatment (mean age: 14 years, range: 2–40 years)	Scoliosis	Lower mean degrees of scoliosis	Glucocorticoid exposure
				Loss of Ambulation	Delay in loss of ambulation	
Balaban et al., 2005 (NR) [47]	Case-control study (Level 4)	DFZ and PDN/PRED	49 patients with DMD treated with DFZ (mean age: 14 years, range: NR) or PDN/PRED (mean age: 15 years, range: NR) or no treatment (mean age: 14 years, range: NR)	Scoliosis	Reduced number of spinal surgeries versus untreated	Glucocorticoid exposure
				Respiratory Health and Function	Improved FVC between 7–15 years old versus untreated	
				Muscle Strength	Grip and pinch strength (maximum hand-held weight which could be lifted overhead) improved in DFZ and PDN/PRED versus untreated	
				Lower Extremity and Motor Function	Improved walk/run 9 metres, STS, and 4SC versus untreated	
Alman et al., 2004 (CA) [48]	Non-randomised controlled cohort study (Level 3)	DFZ	54 patients with DMD treated with DFZ (mean age: 9 years, range: NR) or no treatment (mean age: 9 years, range: NR)	Scoliosis	Decrease in rate of scoliosis > 20 degrees and need for spinal surgery	Glucocorticoid exposure
Lebel et al., 2013 (CA) [49]	Non-randomised controlled cohort study (Level 3)	DFZ	54 patients with DMD treated with DFZ (mean age: 9 years, range: NR) or no treatment (mean age: 9 years, range: NR)	Scoliosis	Decrease in rate of scoliosis > 20 degrees and need for spinal surgery	Glucocorticoid exposure
				Survival	Reduction in mortality	
Kinali et al., 2007 (UK) [50]	Case series (Level 4)	KAFOS; PDN/PRED	123 patients with DMD (mean age: NR, range: NR)	Scoliosis	*KAFOS* Longer duration of use reduces scoliosis severity *PDN/PRED* Later age at scoliosis onset linked to duration of use	Orthoses; and Glucocorticoid exposure
McDonald et al., 2018 (*) [51]	Observational study with dramatic effect (Level 2)	DFZ and PDN/PRED	440 patients with DMD (mean age: NR, range: 2–28 years)	Survival	Reduction in mortality (>1 year of exposure)	Glucocorticoid exposure
				Loss of Ambulation	Delay in loss of ambulation (>1 year of exposure) and favouring DFZ	
				Upper extremity function	Retained hand function as given by Brooke score (>1 year of exposure) and favouring DFZ	
				Lower Extremity and Motor Function	Improved STS (>1 year of exposure) and favouring DFZ	
Ogata et al., 2009 (JP) [52]	Case series (Level 4)	ACE inhibitor (enalapril/lisinopril) and BB (bisoprolol/carvedilol/metoprolol)	52 patients with DMD receiving symptomatic treatment (mean age: 18 years, range: NR) or asymptomatic treatment (mean age: 20 years, range: NR)	Survival	Overall survival improved in the early treatment (asymptomatic) group	Cardiac medication
Rall and Grim, 2012 (DE) [53]	Case-control study (Level 4)	Ventilation support	94 patients with DMD (mean age: NR, range: NR)	Survival	Improved overall survival	Ventilation support
Jeppesen et al., 2003 (DK) [54]	Case-control study (Level 4)	Ventilation support	159 patients with DMD (mean age: NR, range: NR)	Survival	Reduction in all-cause mortality	Ventilation support
Eagle et al., 2007 (UK) [55]	Case-control study (Level 4)	Spinal surgery and ventilation; ventilation no spinal surgery; no spinal surgery or ventilation	100 patients with DMD (mean age: NR, range: NR)	Survival	Spinal surgery/ ventilation and ventilation no spinal surgery improved survival with spinal surgery/ventilation having a larger impact	Ventilation support; and spinal surgery
Eagle et al., 2002 (UK) [56]	Case-control study (Level 4)	Nocturnal ventilation support	183 patients with DMD (mean age: NR, range: NR)	Survival	Reduction in mortality	Ventilation support
Gomez-Merino et al., 2002 (NR) [57]	Case-control study (Level 4)	Non-invasive respiratory aids	91 patients with DMD (mean age: NR, range: NR)	Survival	Prolongation of survival	Ventilation support
Kieny et al., 2013 (FR) [58]	Case-control study (Level 4)	Ventilation support	119 patients with DMD (mean age: NR, range: NR)	Survival	Prolongation of survival	Ventilation support
Ishikawa et al., 2011 (JP) [59]	Case-control study (Level 4)	Non-invasive respirator aids (including mechanically assisted coughing)	187 patients with DMD (mean age: NR, range: NR)	Survival	Prolongation of survival compared to invasive treatment	Ventilation support
Adorisio et al., 2019 (NR) [60]	Case-control study (Level 4)	Left ventricular assist device in combination with cardiac medication and OMT	12 patients with DMD (mean age: NR, range: NR)	Survival	Improved survival	Left ventricular assist device
Davidson et al., 2012 (AU) [61]	Case series (Level 4)	DFZ and PDN/PRED	144 patients with DMD (mean age: NR, range: NR)	Loss of Ambulation	Reduction in risk of loss of ambulation	Glucocorticoid exposure; and DMD mutation type
		Dystrophin gene deletions			Increased risk of loss of ambulation	
Bonifati et al., 2006 (IT) [62]	Non-randomised controlled cohort study (Level 3)	DFZ and PDN/PRED	48 patients with DMD (mean age: NR, range: NR)	Loss of Ambulation	Early treatment initiation and increased treatment duration delay loss of ambulation	Glucocorticoid exposure
Bello et al., 2015 (*) [63] Bello et al., 2015 (*) [64] Bello et al., 2015 (*) (IT) [65]	Observational study with dramatic effect (Level 2)	DFZ and PDN/PRED	340 patients with DMD (283 for the genotype sub-population) (mean age: 16 years, range: 5–33 years)	Loss of Ambulation	Delay in loss of ambulation; DFZ more favourable	Glucocorticoid exposure; and DMD genetic modifiers
		TG/GG genotype at SPP1 rs28357094			Earlier loss of ambulation	
		LTBP4 haplotype			Delayed loss of ambulation	
Bello et al., 2014 (*) [66]	Observational study with dramatic effect (Level 2)	DFZ and PDN/PRED	332 patients with DMD (mean age: NR, range: NR)	Loss of Ambulation	Delay in loss of ambulation, DFZ more favourable	Glucocorticoid exposure; and DMD genetic modifiers
		G allele at SPP1rs28357094			Earlier loss of ambulation	
Bello et al., 2016 (*) [11]	Observational study with dramatic effect (Level 2)	DFZ and PDN/PRED Deletion of exon 3–7 and exon 44 skipping	212 patients with DMD (mean age: NR, range: NR)	Loss of Ambulation	Delay in loss of ambulation	Glucocorticoid exposure; and DMD mutation type
Bello et al., 2016 (*) [67]		Exon 44 skipping				DMD mutation type
Goemans et al., 2019 (*) [68] Goemans et al., 2019 (*) [69]	Case series (Level 4)	DFZ and PDN/PRED	85 patients with DMD (mean age: 9 years, range: NR)	Loss of Ambulation	Predictive of loss of ambulation	Glucocorticoid exposure; greater weight; lower height; and lower BMI (in combination)
Kim et al., 2015 (US) [70]	Observational study with dramatic effect (Level 2)	DFZ and PDN/PRED	477 patients with DMD (mean age: NR, range: NR)	Loss of Ambulation	Delay in loss of ambulation with larger effect for those treated longer in the <11 year olds	Glucocorticoid exposure
Schara et al., 2001 (DE) [71]	Case-control study (Level 4)	DFZ	13 patients with DMD (mean: NR, range: 9–18 years)	Loss of Ambulation	Delay in loss of ambulation	Glucocorticoid exposure
				Respiratory Health and Function	Improved FVC	
				Muscle Strength	Improved muscle strength as given by MRC scale	
				Lower Extremity and Motor Function	Improved Vignos functional score, STS, 4SC, and walking ability	
Van den Bergen et al., 2014 (NL) [72]	Retrospective observational study (Level 2)	Glucocorticoids (drug NR)	336 patients with DMD (mean age: NR, range: NR)	Loss of Ambulation	Delay in loss of ambulation	Glucocorticoid exposure
Van den Bergen et al., 2014 (NL) [73]	Case control study (Level 4)	Glucocorticoids (drug NR) Exon 44 (vs. 45, 51, and 53)	114 patients with DMD (mean age: NR, range: NR)	Loss of Ambulation	Delay in loss of ambulation	Glucocorticoid exposure; and DMD mutation type
Wang et al., 2014 (US) [74]	Online survey (Level 5)	DFZ and PDN/PRED	1,057 patients with DMD (mean age: NR, range: NR)	Loss of Ambulation	Delay in loss of ambulation with DFZ favourable over PDN/PRED	Glucocorticoid exposure
		Age at diagnosis			Delay in loss of ambulation	Age at diagnosis
Ricotti et al., 2012 (UK) [75] Ricotti et al., 2011 (UK) [76] Ricotti et al., 2011 (UK) [77]	Case series (Level 4)	PDN/PRED	334–400 patients with DMD (mean age: NR, range: 3–15 years)	Loss of Ambulation	Delay in loss of ambulation in daily PDN-treated compared to intermittent PDN	Glucocorticoid exposure
DeSilva et al., 1987 (US) [78]	Non-randomised controlled cohort study (Level 3)	PDN/PRED	54 patients with DMD (mean age: NR, range: NR)	Loss of Ambulation	Delay in loss of ambulation	Glucocorticoid exposure
Yilmaz et al., 2004 (TR) [79] Yilmaz et al., 2004 (TR) [80] Tunca et al., 2001 (TR) [81]	Historically controlled cohort study (Level 4)	PDN/PRED	88 patients with DMD treated with PDN/PRED (mean age: 7 years, range: 3–11 years) or no treatment (mean age: 7 years, range: 5–9 years)	Loss of Ambulation	Delay in loss of ambulation	Glucocorticoid exposure
				Lower Extremity and Motor Function	Improved 10WRT at 6 months	
Yilmaz et al., 2004 (TR) [79] Yilmaz et al., 2004 (TR) [80]				Muscle Strength	Improved muscle strength as given by Lovett’s tests	
Biggar et al., 2001 (CA) [82]	Case control (Level 4)	DFZ	54 patients with DMD (mean age: NR, range: NR)	Loss of Ambulation	Delay in loss of ambulation	Glucocorticoid exposure
				Respiratory Health and Function	Improved FVC	
				Lower Extremity and Motor Function	Improved 4SC and STS	
Ciafaloni et al., 2013 (US) [83] Ciafaloni et al., 2016 (US) [84]	Observational study with dramatic effect (Level 2)	Age at onset of symptoms	825 patients with DMD (mean age: NR, range: NR)	Loss of Ambulation	Earlier loss of ambulation for earlier symptom development	Age at onset of symptoms
Bello et al., 2016 (*) [85]	Genome-wide association study (Level 4)	Minor allele at rs1883832	109 patients with DMD (mean age: NR; range: NR)	Loss of Ambulation	Delay in loss of ambulation	DMD genetic modifiers
Haber et al., 2021 (US) [86]	Case control study (Level 4)	Exon 8 and Exon 44 skip deletions	358 patients with DMD (mean age: NR; range: NR)	Loss of Ambulation	Delay in loss of ambulation	DMD mutation type
Mercuri et al., 2020 (NR) [87]	Non-randomised controlled study (Level 3)	ATA compared to external controls	181 patients with DMD (mean age: NR, range: NR) or external control (mean age: NR, range: 2–28 years)	Loss of Ambulation	Delay in loss of ambulation	ATA treatment
				Lower Extremity and Motor Function	Improved STS and 4SC	
Wang et al., 2018 (*) [88]	Case series (Level 4)	Glucocorticoids; DMD mutation type	765 patients with DMD (mean age: NR; range: NR)	Loss of Ambulation	Delay in loss of ambulation: Glucocorticoids, exon 44, exon 3–7, exon 45, exon 8 Earlier loss of ambulation: Exon 51, exon 49–50	Glucocorticoid exposure; DMD mutation type
Forst et al., 1995 [89] (GER)	Observational study with dramatic effect (Level 2)	Lower limb surgery	213 patients with DMD (mean age: 6.56 years; range: 4.02–8.26)	Loss of Ambulation	Delay in loss of ambulation	Lower limb surgery
Forst et al., 1995 [90] (GER)	Observational study with dramatic effect (Level 2)	Lower limb surgery	123 patients with DMD (mean age: NR; range: NR)	Loss of Ambulation	Delay in loss of ambulation	Lower limb surgery
Servais et al., 2015 (FR) [91]	Case-control study (Level 4)	Exon 53	53 patients with DMD (DMD 53: mean age: 13.9, range: NR or DMD-all-non-53: mean age: 14 years, range: NR or DMD-del-non-53: mean age: 14.1, range: NR)	Loss of Ambulation	Delay in loss of ambulation compared to DMD-all-non-53 and DMD del-non-53	DMD mutation type
				Cardiac Health and Function	Lower LVEF and higher contracture score compared to DMD-del-non-53	
				Muscle Strength	Lower pinch strength in exon 53 compared to DMD-all-non-53	
Escolar et al., 2011 (US) [92]	Randomised controlled trial (Level 2)	PDN/PRED (daily dose with PLC at weekend; weekend dose with PLC during weekdays)	64 patients with DMD (mean age: 7 years, range: NR)	Respiratory Health and Function	Weekend dosing equivalent to daily dosing as given by MVV; MIP	Glucocorticoid exposure
				Upper Extremity Function	Weekend dosing equivalent to daily dosing as given by Brooke score	
				Muscle Strength	Weekend dosing equivalent to daily dosing as given by QMT and MMT	
				Lower Extremity and Motor Function	Weekend dosing equivalent to daily dosing as given by STS, 4SC and 10WRT	
Tachas et al., 2020 (NR) [93]	Randomised trial (Level 2)	ATL1102 compared to external natural history control	29 patients with DMD (mean age: 14.9 years, range: 12–18 years) or external control (mean age: 15.61, range: NR)	Upper Extremity Function	Improved upper limb function as given by PUL	ATL1102 treatment
Daftary et al., 2007 (US) [94]	Case-control study (Level 4)	DFZ and PDN/PRED	35 patients with DMD (mean age: NR, range: 7–21 years)	Respiratory Health and Function	Long-term glucocorticoid therapy improves PCF and MEP	Glucocorticoid exposure
Abresch et al., 2013 (*) [95]	Case-control study (Level 4)	DFZ and PDN/PRED	341 patients with DMD (mean age: NR, range: 6–28 years)	Respiratory Health and Function	Improved MIP, MEP and PCF	Glucocorticoid exposure
Henricson et al., 2013 (*) [96]	Case series (Level 4)	DFZ and PDN/PRED (current users vs. naïve users)	340 patients with DMD (mean age: NR, range: 2–28 years)	Respiratory Health and Function	Improved FVC; MIP; PEFR; FEV_1_	Glucocorticoid exposure
				Upper Extremity Function	Improved Brooke score	
				Lower Extremity and Motor Function	Improved Vignos, STS, 4SC, and 10WRT	
McDonald et al., 2018 (*) [97]	Case control study (Level 4)	DFZ and PDN/PRED	397 patients with DMD (median: 9 years, IQR: 2–28 years)	Respiratory Health and Function	Improved FVC	Glucocorticoid exposure
Henricson et al., 2017 (US) [98] McDonald et al., 2017 (US) [99]	Case control (Level 4)	DFZ and PDN/PRED	233 patients with DMD (mean age: 13 years, range: 6–28 years)	Respiratory Health and Function	Sustained FVC and PEFR	Glucocorticoid exposure
Ricotti et al., 2011 (UK) [77]	Case series (Level 4)	PDN/PRED	334–400 patients with DMD (mean age: NR, range: 3–15 years)	Respiratory Health and Function	Sustained FVC in daily PDN	Glucocorticoid exposure
Pradhan 2006 (IN) [100]	Non-randomised controlled cohort study (Level 3)	PDN/PRED	34 patients with DMD (mean age: NR, range: NR)	Respiratory Health and Function	Improved short-term PEFR	Glucocorticoid exposure
				Muscle Strength	Improved MRC	
Fenichel et al., 1991 (US) [101]	RCT (Level 2)	PDN/PRED	103 patients with DMD (mean age: NR, range: 5–15 years)	Respiratory Health and Function	Daily and alternate day PDN/PRED improved FVC and MVV at 12 months	Glucocorticoid exposure
				Muscle Strength	Daily and alternate day PDN/PRED improved muscle strength using an unspecified measure at 6 months but more sustained with daily Both doses improved muscle mass as given by creatinine excretion	
				Lower Extremity and Motor Function	Daily and alternate day PDN/PRED improved STS and 4SC	
Dubow et al., 2016 (NR) [102]	RCT (Level 2)	DFZ and PDN/PRED	45 patients with DMD (mean age: NR, range: NR)	Respiratory Health and Function	1.2 mg/kg/day dose of DFZ versus PLC improves MVV	Glucocorticoid exposure
Comi et al., 2017 (*) [103] McDonald et al., 2016 (*) [104]	Historically-controlled study (Level 4)	ATA	167 patients with DMD (mean age: 16 years, range: NR)	Respiratory Health and Function	Improved FVC	ATA treatment
Kelley et al., 2019 (*) [105]	Case series (Level 4)	Gly16 ADRB2 polymorphism	175 patients with DMD (mean age: NR, range: 3–25 years)	Respiratory Health and Function	*Gly16 genotype* 6.52X likelier of receiving nocturnal ventilation compared to Arg16 *Patient weight* Predictor of need for nocturnal ventilation	DMD genetic modifier; weight
Angliss et al., 2020 (AU) [106]	Case control (Level 4)	Ventilation	29 patients with DMD (median: 14.66; IQR: NR)	Respiratory Health and Function	FVC improved in steroid naïve but accelerated decline in steroid users	Ventilation support
Bello et al., 2020 (IT) [107]	Case control (Level 4)	DMD mutation type and DMD genetic modifiers; Glucocorticoids	327 patients with DMD (mean age: 11.7, range: NR)	Respiratory Health and Function	*Exon 44 3’ mutation*: Lower FVC, lower FEV1 and lower PEF *Glucocorticoid* Increased FVC, FEV1 and PEF *Skip 51*, *Skip 53* Decreased FVC, decreased FEV1, decreased PEF *Splice site*, *Skip 8*, *Skip 44* Increased FVC Skip 8, splice site Increased FEV1, increased PEF *Nonsense mutation* Decreased FVC and FEV1 *Dominant G genotype at rs28357094 in the SPP1 promoter* Reduced FVC and PEF *Additive T genotype at rs1883832 in the CD40 5’ untranslated region* Reduced FVC, FEV1 and PEF	Glucocorticoid exposure; DMD mutation type; DMD genetic modifiers
Iff et al., 2020 (US) [108]	Case control (Level 4)	ETEP versus untreated controls	283 patients with DMD (mean age: 14.1 years, range: NR)	Respiratory Health and Function	Attenuates respiratory function (indirectly measured)	ETEP exposure
McDonald et al., 2020 (*) [109] McDonald et al., 2020 (*) [110]	Randomised trial (Level 2)	ATA versus external natural history control	95 patients with DMD (mean age: NR, range: NR)	Respiratory Health and Function	Delay in respiratory decline as given by FVC	ATA treatment
				Loss of Ambulation	Delay in loss of ambulation	
Buyse et al., 2011 (BE) [111]	Randomised trial (Level 2)	IDE and PLC	21 patients with DMD (mean age: NR, range: 8–16 years)	Respiratory Health and Function	Improved PEF	IDE treatment
				Cardiac Health and Function	Improved peak systolic radial strain in the LV inferolateral wall	
Karafilidis et al., 2018 (NR) [112]	Randomised trial (Level 2)	IDE and PLC	64 patients with DMD (mean age: NR, range: 10–18 years)	Respiratory Health and Function	Improved PEF and FEV1	IDE treatment
Khan et al., 2019 (NR) [113] Khan et al., 2019 (NR) [114] Khan et al., 2019 (NR) [115]	Randomised trial (Level 2)	ETEP and natural history control	414 patients with DMD (mean age: NR, range: 7–16 years) or natural history control (mean age: NR, range: 2–28 years)	Respiratory Health and Function	Reduced decline in respiratory decline as given by percent predicted FVC	ETEP treatment
Mendell et al., 2014 (NR) [116] Mendell et al., 2014 (NR) [117] Mendell et al., 2014 (NR) [118]	Randomised trial (Level 2)	ETEP and PLC	12 patients with DMD (median age: 9.7 years, IQR: NR; range: 7–13 years)	Respiratory Health and Function	Improved MEP and FVC	ETEP treatment
Mendell et al., 2014 (NR) [119] Mendell et al., 2014 (NR) [120] Mendell et al., 2015 (NR) [121] Kaye et al., 2014 (NR) [122] Kaye et al., 2015 (NR) [123] Kaye et al., 2015 (NR) [124] Kaye et al., 2015 (NR) [125]				Lower Extremity and Motor Function	Improved 6MWT	
Mendell et al., 2021 (NR) [126] Mendell et al., 2016 (NR) [127] Mendell et al., 2017 (NR) [128] Mendell et al., 2016 (NR) [129] Mendell et al., 2016 (NR) [130]	Randomised controlled trial (Level 2)	ETEP compared to external controls	12 patients with DMD (mean age: 9.4 years, range: 7–13 years) or no treatment (mean age: 9.6 years, range: 7–13 years)	Loss of Ambulation	Delay in loss of ambulation	ETEP treatment
				Lower Extremity and Motor Function	Improved 6MWT	
McDonald et al., 2020 (*) [131]	Randomised trial (Level 2)	Analysis of PLC arm data; DFZ and PDN/PRED	115 patients with DMD (mean age: NR, range: 7–14 years)	Lower Extremity and Motor Function	Improved 4SC, 6MWT, STS and NSAA	Glucocorticoid exposure
Lawrence et al., 2018 (NR) [132]	Randomised trial (Level 2)	IDE and PLC	23 patients with DMD (mean age: NR, range: 10–18 years)	Respiratory Health and Function	Improvement in respiratory function as given by reduced bronchopulmonary adverse events	IDE treatment
Rummey et al., 2018 (NR) [133]	Follow-up study (Level 3)	IDE and PLC	64 patients with DMD (mean age: 14.3 years, range: 10–18 years)	Respiratory Health and Function	Improved PEF	IDE treatment
Kanazawa et al., 1991 (JP) [134]	Follow-up study (Level 3)	cDMD deficit	24 patients with DMD (mean age: 14.2 years; range: NR) or non-deficit group: mean age: 14.7 years, range: NR)	Respiratory Health and Function	Worse pulmonary function	DMD mutation type
Hussein et al., 2006 (EG) [135]	Case-control (Level 4)	PDN/PRED	18 patients with DMD (mean age: 5 years, range: NR)	Muscle Strength	Improvement in muscle strength as given by MRC scale	Glucocorticoid exposure
Angelini et al., 1994 (IT) [136]	RCT (Level 2)	DFZ	28 patients with DMD treated with DFZ (mean age: 8 years, range: NR) or PLC (mean age: 8 years, range: NR)	Muscle Strength	Improvement in muscle strength as given by MRC scale (>1 year of treatment)	Glucocorticoid exposure
				Lower Extremity and Motor Function	Improved STS	
Fenichel et al., 1991 (US) [137]	Historically-controlled study (Level 4)	PDN/PRED	92 patients with DMD (mean age: NR, range: 5–15 years)	Muscle Strength	Improved muscle strength using an unspecified measure versus controls Improved more for >0.65mg/kg dose compared to <0.65mg/kg	Glucocorticoid exposure
Hu et al., 2015 (CN) [138]	RCT (Level 2)	PDN/PRED	66 patients with DMD (mean age: NR, range: 4–12 years)	Muscle Strength	Stabilised MRC	Glucocorticoid exposure
				Lower Extremity and Motor Function	Improved 10WRT, 4SC, and STS	
Rifai et al., 1995 (US) [139]	Case-control (Level 4)	PDN/PRED	6 patients with DMD (mean age: NR, range: 5–8 years)	Muscle Strength	Improved muscle strength and mass as given by MMT, QMT, and creatinine excretion)	Glucocorticoid exposure
Backman and Henriksson, 1995 (SE) [140]	RCT (Level 2)	PDN/PRED	37 ambulatory (mean age: 8 years, range: 4–11 years) or non-ambulatory (mean age: 13 years, range: 8.0–19 years) patients with DMD	Muscle Strength	Improved muscle strength as given by grip strength (strain gauge) and myometric evaluation	Glucocorticoid exposure
				Lower Extremity and Motor Function	Scott functional testing improved during first 3 months of treatment	
				Upper Extremity Function	Brooke score improved during first 3 months of treatment	
Connolly et al., 2002 (US) [141]	Historically controlled cohort study (Level 4)	PDN/PRED	42 patients with DMD (mean age: NR, range: NR)	Muscle Strength	Improvement in grip (Jamar grip meter) and upper extremity strength using a myometry	Glucocorticoid exposure
				Lower Extremity and Motor Function	STS, walk/run 9m, and 4SC improved in younger boys versus older boys	
Griggs et al., 1993 (CA/US) [142]	RCT (Level 2)	PDN/PRED	107 patients with DMD (mean age: NR, range: 5–15 years)	Muscle Strength	Improved muscle strength as given by muscle mass increases (creatinine excretion), myometric evaluation and MMT Larger improvement in 075mg/kg versus 0.30mg/kg	Glucocorticoid exposure
Mesa et al., 1991 (AR) [143]	Non-randomised controlled study (Level 3)	DFZ	28 patients with DMD (mean age: NR, range: 5–11 years)	Muscle Strength	Improvement in muscle strength as given by myometric evaluation	Glucocorticoid exposure
				Lower Extremity and Motor Function	Improved Scott functional score and STS	
Beenakker et al., 2005 (NL) [144]	RCT (Level 2)	PDN/PRED	17 patients with DMD (mean age: 6 years, range: NR)	Muscle Strength	Intermittent PDN/PRED improves total muscle force as given by myometric evaluation	Glucocorticoid exposure
				Lower Extremity and Motor Function	Intermittent PDN/PRED improves 9 metre run/walk and 4SC	
Griggs et al., 1991 (CA/US) [145]	RCT (Level 2)	PDN/PRED	99 patients with DMD (mean age: NR, range: NR)	Muscle Strength	Improved muscle strength as given by myometric evaluation and MMT. Improvements larger in 075mg/kg versus 0.30mg/kg	Glucocorticoid exposure
				Lower Extremity and Motor Function	9m run/walk test and STS improved in 0.75mg/kg; 4SC improved in both 0.75mg/kg and 0.30mg/kg	
				Respiratory Health and Function	Improved FVC versus PLC at both 0.3 and 0.75mg/kg	
Merlini et al., 2003 (IT) [146]	Case-control study (Level 4)	PDN/PRED	8 patients with DMD treated with PDN/PRED (mean age: 4 years, range: NR) or no treatment (mean age: 4 years, range: NR)	Muscle Strength	Improved muscle strength as given by myometric evaluation but only in the leg megascore	Glucocorticoid exposure
				Lower Extremity and Motor Function	Improved STS	
Pegoraro et al., 2011 (IT) [147]	Historically controlled cohort study (Level 4)	SPP1 genotype	262 patients with DMD (mean age: NR, range: NR)	Muscle Strength	G allele leads to weaker MRC scores and lower grip strength	DMD genetic modifiers
Fenichel et al., 2001 (NR) [148]	Randomised trial (Level 2)	OXAN vs PLC	51 patients with DMD (mean age: NR, range: 5–10 years)	Muscle Strength	Improved muscle strength score using an unspecified measure	OXAN treatment
Fenichel et al., 1997 (US) [149]	Case-series (Level 4)	OXAN	10 patients with DMD (mean age: NR, range: 6–9 years)	Muscle Strength	Improved muscle strength as given by manual muscle testing	OXAN treatment
Campbell et al., 2020 (*) [150]	Meta-analysis (Level 1)	ATA and PLC	342 patients with DMD (mean age: NR; range: 8.3–9.0)	Lower Extremity and Motor Function	Improved 6MWD, 4SC and 10WRT	ATA treatment
Chesshyre et al., 2020 (ENG) [151]	Case series (Level 4)	Dp140 deletion	320 patients with DMD (mean age: MR; range: NR)	Lower Extremity and Motor Function	Lower NSAA	DMD genetic modifiers
Clemens et al., 2020 (US and CAN) [152]	Randomised trial (Level 2)	Vitlolarsen (low dose and high dose)	16 patients with DMD (mean age: 7.4; range: NR)	Lower Extremity and Motor Function	Improved 10WRT, 6MWT, STS and NSAA	VIT treatment
Finkel et al., 2021 (NR) [153] Finkel et al., 2018 (NR) [154] Finkel et al., 2019 (NR) [155] Finkel et al., 2019 (NR) [156] Sweeney et al., 2019 (US) [157]	Randomised trial (Level 2)	EDASA and PLC	31 patients with DMD (mean age: 6.1; range: 4–7)	Muscle Strength	Improved lower leg muscle health as given by MRI transverse relaxation time constant	EDASA treatment
Parreira et al., 2010 (NR) [158]	Case series (Level 4)	DFZ and PDN/PRED	90 patients with DMD (mean age: NR, range: 5–12 years)	Muscle Strength	Delay in decline in muscle strength as given by MRC index	Glucocorticoid exposure
Willcocks et al., 2013 (NR) [159]	Follow-up study (Level 3)	DFZ and PDN/PRED	145 patients with DMD (mean age: NR, range: 5–14 years)	Muscle Strength	Delays decline in muscle as given by MRI and MRS transverse relaxation time constant	Glucocorticoid exposure
Goemans et al., 2020 (NR) [160]	Case series (Level 4)	DFZ	316 patients with DMD (median age: 7.9 years, range 4.4–19.4 years)	Lower Extremity and Motor Function	Delay loss of STS	Glucocorticoid exposure
Goemans et al., 2020 (NR) [161]	Historically controlled study (Level 4)	Glucocorticoid, height, weight, BMI	371 patients with DMD (mean age: NR; range: 8.81 and 9.36)	Lower Extremity and Motor Function	Glucocorticoid, including duration, height, weight and BMI predictive of 4SC	Glucocorticoid exposure, height, weight, BMI
Wilton et al., 2013 (US) [162]	Randomised trial (Level 2)	ETEP and PLC	NR patients with DMD (mean age: NR, range: NR)	Lower Extremity and Motor Function	Improvements in 6MWT	ETEP treatment
Signorovitch et al., 2017 (*) [163] Signorovitch et al., 2019 (*) [164] Signorovitch et al., 2019 (*) [165] Signorovitch et al., 2019 (*) [166]	MA (Level 1)	DFZ and PDN/PRED	231 patients with DMD (mean age: NR, range: NR)	Lower Extremity and Motor Function	DFZ improved NSAA, 6MWT, STS, and 4SC compared to PDN/PRED	Glucocorticoid exposure
Gupta et al., 2020 (UK) [167]	Case series Level 4)	Glucocorticoids (drug NR)	465 patients with DMD (mean age: NR, range: NR)	Lower Extremity and Motor Function	Improved NSAA compared to steroid-naïve	Glucocorticoid exposure
Goemans et al., 2016 (NR) [168] Goemans et al., 2016 (NR) [169]	Open-label study (Level 2)	DRIS and natural history control	12 patients with DMD (mean age: 9.9 years, range: NR) or natural history control (mean age: 9.4 years, range: NR)	Lower Extremity and Motor Function	Improvement in 6MWT	DRIS treatment
Ricotti et al., 2013 (UK) [170] Ricotti et al., 2012 (UK) [75] Ricotti et al., 2011 (UK) [76] Ricotti et al., 2011 (UK) [77]	Case series (Level 4)	PDN/PRED	334–400 patients with DMD (mean age: NR, range: 3–15 years)	Lower Extremity and Motor Function	Improved NSAA in daily PDN-treated compared to intermittent PDN	Glucocorticoid exposure
Schreiber et al., 2018 (FR) [171] Schreiber et al., 2015 (FR) [172] Schreiber et al., 2016 (FR) [173]	Case-control study (Level 4)	DFZ and PDN/PRED	74–76 patients with DMD treated with DFZ and PDN/PRED (mean age: 8 years, range: 6–11 years) or no treatment (mean age: 8 years, range: 6–12 years)	Lower Extremity and Motor Function	Improved muscle function measure	Glucocorticoid exposure
Alfano et al., 2019 (US) [174]	Non-randomised controlled study (Level 3)	DFZ and PDN/PRED	148 patients with DMD (mean age: NR, range: 3–16 years)	Lower Extremity and Motor Function	Improved 10WRT and 100m walking ability	Glucocorticoid exposure
Goemans et al., 2016 (BE) [175]	Case series (Level 4)	DFZ and PDN/PRED	39 patients with DMD (mean age: 9 years, range: 4–16 years)	Lower Extremity and Motor Function	Improved 6MWD including duration of use; those with lower 6MWD showed larger declines	Glucocorticoid exposure
					Increased height and weight produced larger declines in 6MWD	Height; and weight
Goemans et al., 2018 (BE) [176]	Case series (Level 4)	DFZ and PDN/PRED	81 patients with DMD (mean age: 10 years, range: NR)	Lower Extremity and Motor Function	Improved 4SC including duration of use	Glucocorticoid exposure
Mazzone et al., 2014 (NR) [177]	Non-randomised controlled study (Level 3)	DFZ and PDN/PRED	96 patients with DMD (mean age: NR, range: NR)	Lower Extremity and Motor Function	Improved 6MWT; baseline 6MWT >350m showed larger improvements	Glucocorticoid exposure
Shieh et al., 2018 (NR) [178] Shieh et al., 2018 (NR) [178] Darras et al., 2018 (NR) [179] [NR]	Meta-analysis (Level 1)	DFZ and PDN/PRED	147 patients with DMD (mean age: NR, range: NR)	Lower Extremity and Motor Function	Improved 6MWT favouring DFZ	Glucocorticoid exposure
Bushby et al., 2014 (*) [180] Mah et al., 2011 (*) [181] McDonald et al., 2013 (*) [182] McDonald et al., 2014 (*) [183] McDonald et al., 2014 (*) [184]	Randomised trial (Level 2)	ATA	174 patients with DMD (median age: 8 years, IQR: 5–20 years)	Lower Extremity and Motor Function	Low dose ATA improved 6MWT including larger improvements in baseline 6MWT <350m	ATA treatment
McDonald et al., 2017 (*) [185]	Randomised trial (Level 2)	ATA	230 patients with DMD treated with ATA (mean age: 9 years, range: 7–10 years) or PLC (mean age: 9 years, range: 8–10 years)	Lower Extremity and Motor Function	Improved 6MWT in 300-400m baseline 6MWT sub-group	ATA treatment
McDonald et al., 2019 (*) [186] McDonald et al., 2019 (*) [187] Bushby et al., 2016 (NR) [188]	Randomised trial (Level 2)	ATA	228 patients with DMD (mean age: NR, range: NR)	Lower Extremity and Motor Function	Preserved NSAA	ATA treatment
McDonald et al., 2017 (*) [189] McDonald et al., 2018 (*) [190] McDonald et al., 2018 (*) [191]	Randomised trial (Level 2)	ATA	168 patients with DMD (mean age: NR, range: NR)	Lower Extremity and Motor Function	Improved 6MWT, 4SC, and 10WRT	ATA treatment
Mercuri et al., 2019 (NR) [192] Muntoni et al., 2019 (NR) [193]	Non-randomised controlled study (Level 3)	ATA versus external natural history control	187 patients with DMD (mean age: NR, range: NR)	Lower Extremity and Motor Function	Improved STS and 4SC	ATA treatment
				Loss of Ambulation	Loss of Ambulation	
Brogna et al., 2019 (*) [194] Brogna et al., 2019 (*) [195]	Case series (Level 4)	Skip exons 44, 45, 51, and 53	92 patients with DMD (mean age: 8 years, range: NR)	Lower Extremity and Motor Function	Exon skipping impacts 6MWT	DMD mutation type
Komaki et al., 2020 (JP) [196]	Randomised trial (Level 2)	TAS-205 and PLC	36 patients with DMD (mean age: 8.3, range: NR)	Lower Extremity and Motor Function	High dose improves muscle volume index	TAS-205 treatment
Hoffman et al.., 2019 (NR) [197]	Randomised non-controlled trial (Level 3)	VAM	48 patients with DMD (mean age: NR; range: 4–7 years)	Lower Extremity and Motor Function	Improved 10WRT, STS, 6MWT	VAM treatment
Smith et al., 2020 (*) [198]	Historically controlled study (Level 4)	VAM and external natural history control	122 patients with DMD (mean age: NR, range: 4–7 years)	Lower Extremity and Motor Function	Improved STS, 4SC, NSAA, 10WRT	VAM treatment
Koeks et al., 2017 (*) [199]	Case series (Level 4)	Glucocorticoid exposure	5345 patients with DMD (mean age: NR, range: NR)	Loss of Ambulation	Delay in loss of ambulation	Glucocorticoid exposure; DMD mutation type
				Scoliosis	Reduced scoliosis	
				Respiratory Health and Function	Reduced need for ventilation	
				Cardiac Health and Function	Reduced cardiomyopathy	
		Exon 45 deletion		Loss of Ambulation	Delay in loss of ambulation	
Voit et al., 2014 (*) [200]	Randomised trial (Level 2)	DRIS and PLC	53 patients with DMD (DRIS continuous: mean age: 7.2 years, range: NR and DRIS intermittent: mean age: 7.7 years) or PLC (mean age: 6.9 years, range: NR)	Lower Extremity and Motor Function	Improved STS versus PLC for both continuous and intermittent DRIS. 6MWD was improved in the continuous regimen versus PLC at week 25	DRIS treatment
McDonald et al., 2015 (NR) [201] McDonald et al., 2014 (NR) [202]	Randomised trial (Level 2)	DRIS	535 patients with DMD (mean age: NR, range: NR)	Lower Extremity and Motor Function	Improvement in 6MWT	DRIS treatment
Mayer et al., 2017 (*) [203]	Randomised trial (Level 2)	IDE and PLC	64 patients with DMD (mean age: NR, range: 10–19 years)	Respiratory Health and Function	Reduced decline in pulmonary function as given by FVC	IDE treatment

Note: Argentina (AR). Australia (AU). Belgium (BE). Canada (CA). China (CN). Denmark (DK). Egypt (EG). France (FR). Germany (DE). Holland (NL). India (IN). Italy (IT). Japan (JP). Korea (KR). Not reported (NR). Sweden (SE). Turkey (TR). United Kingdom (UK). United States of America (US). Angiotensin-converting enzyme (ACE). Angiotensin receptor blocker (ARB). Ataluren (ATA). Best standard of care (BSC). Beta2-adrenergic receptor (ADRB2). Beta blocker (BB). Body mass index (BMI). Cluster of differentiation 40 (CD40). Deflazacort (DFZ). Drisapersen (DRIS). Duchenne muscular dystrophy (DMD). Dystrophin protein 140 (Dp140). Edasalonexent (EDASA). End systolic volume (ESV). Eplerenone (EPL). Eteplirsen (ETEP). Forced expiratory volume in 1 second (FEV_1_). Four Stair Climb (4SC). Idebenone (IDE). Interquartile range (IQR). Knee-ankle-foot-orthoses (KAFOS). Latent transforming growth factor beta-binding protein 4 (LTBP4). Left ventricular ejection fraction (LVEF). Left ventricular end diastolic dimension (LVEDd). Left ventricular end systolic dimension (LVESd). Left ventricular fractional shortening (LVFS). Left ventricular myocardial performance index (LVMPI). Manual muscle testing (MMT). Maximum expiratory pressure (MEP). Maximum inspiratory pressure (MIP). Maximum voluntary ventilation (MVV). Medical Research Council (MRC). Meridional wall stress (mWS). Meta-analysis (MA). NorthStar Ambulatory Assessment (NSAA). Not applicable (N/A). Optimal Medical Treatment (OMT). Peak cough flow (PCF). Peak expiratory flow rate (PEFR). Peak expiratory flow (PEF). Performance of Upper Limb (PUL). Placebo (PLC). Prednisone (PDN). Prednisolone (PRED). Quantitative muscle testing (QMT). Randomised controlled trial (RCT). Secreted phosphoprotein 1 (SPP1). Single nuclear polymorphisms (SNPs). Six-Minute Walk Test (6MWT). Supine-to-Stand (STS). Ten Metre Walk/Run Test (10WRT). Velocity of circumferential fibre shortening (VCFc). Vamolorone (VAM). Vitlolarsen (VIT).

† OCEBM Level of Evidence.

‡ Indicators with a significant impact on listed disease progression outcome measures.

* Multi-national.

**Fig 2:**
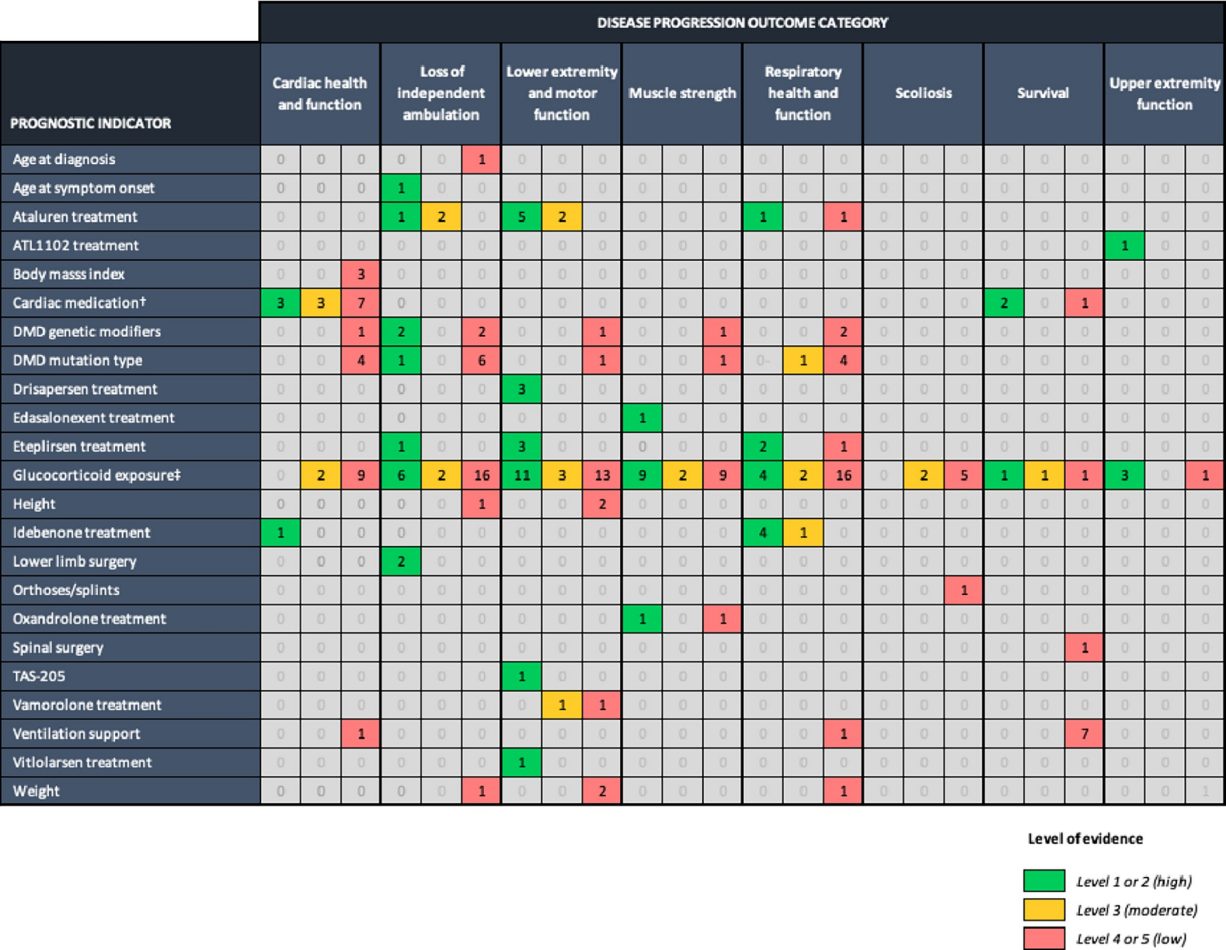
Evidence of prognostic indicators of disease progression in DMD. Note: Numbers shown in the coloured squares refer to the number of studies reporting of the specific indicator. † Angiotensin-converting enzyme (ACE) inhibitors, beta blockers, and/or diuretics. ‡ Age at treatment initiation, dose, duration of exposure, pharmacological agent, and regimen. Duchenne muscular dystrophy (DMD).

We identified a total of 23 prognostic indicators of disease progression in DMD. Endogenous indicators included age at diagnosis, age at onset of symptoms, DMD genetic modifiers, DMD mutation type, height, weight and body mass index (BMI). Exogenous indicators included ataluren treatment, ATL1102, cardiac medication, drisapersen, edasalonexent, eteplirsen, glucocorticoid exposure (including age at glucocorticoid treatment initiation, dose, duration of exposure, pharmacological agent, and regimen), idebenone, lower limb surgery, orthoses, oxandrolone, spinal surgery, TAS-205, vamorolone, vitlolarsen, and ventilation support. The evidence for these prognostic indicators across the pre-defined outcome categories is summarised below and illustrated in Fig 2.

### 3.1. Cardiac health and function

We identified 29 studies presenting evidence of prognostic indicators of disease progression in DMD measured in terms of cardiac health and function [17–45, 91, 111, 199]. In total, seven prognostic indicators were identified: BMI, cardiac medication, DMD genetic modifiers, DMD mutation type, glucocorticoid exposure, idebenone and ventilation support (Table 2). Angiotensin-converting enzyme (ACE) inhibitors, including timing of treatment initiation, have been shown to be significantly associated with improved left ventricular ejection fraction (LVEF) [CEBM Evidence Level 2] [29, 32–34, 42–44], and left ventricular end diastolic and systolic dimension (LVEDd/LVESd) [Level 2]; [30, 31, 45] and left ventricular free wall systolic myocardial velocity [Level 2] [30], beta blockers, when administered in combination with ACE inhibitors, with improved LVEF [Level 4] [32–35], left ventricular fractional shortening (LVFS) [Level 2] [31], LVEDd and LVESd [Level 2] [35], left ventricular myocardial performance index (LVMPI) [Level 4] [35], and left ventricular sphericity index [Level 4];[35] beta blockers with reduced heart failure and arrhythmia [Level 3] [37], and improved LVMPI [Level 2]; [30] timing of unspecified cardiac medication with later onset of cardiomyopathy [Level 4]; [38] eplerenone (EPL) with improved left ventricular systolic strain, LVEF, and end systolic volume (ESV) [Level 2]; [36] and ventilation support in combination with cardiac medication with decreased LVEF and left atrium diameter [Level 4] [39]. Glucocorticoid exposure has been shown to be significantly associated with improved LVEF [Level 4] [17–19, 21, 22, 25], LVFS [Level 3] [17–19, 25–27], LVEDd [Level 4] [19, 25, 26], meridional wall stress (mWS) [Level 4] [26], stabilisation of velocity of circumferential fibre shortening (VCFc) [Level 4] [26], reduction in cardiomyopathy [Level 4] [18, 20, 25, 199], and increases in summed rest score [Level 3] [24], as well as increased risk of cardiomyopathy [Level 4] [28], and decline in LVEF [Level 4] [23] linked to duration of glucocorticoid exposure. Idebenone improves peak systolic radial strain in the LV inferolateral wall [Level 2] [111]. BMI is prognostic of cardiomyopathy [Level 4] [41]. Finally, mutations in exons 51 and 52, as well as latent transforming growth factor beta-binding protein 4 (LTBP4), have been shown to be significantly associated with improved or sustained cardiac health and function [Level 4];[21, 22, 35]; mutations in exons 12, 14, 15, 16, and 17 with increased risk of cardiomyopathy [Level 4] [35]. and deletions in exon 53 with lower LVEF and higher contracture score compared with deletions not treatable by exon 53 skipping [Level 4] [91]. The ACTN3 null genotype is associated with earlier onset of cardiac dysfunction specifically, lower LV dilation-free rate [Level 4] [40].

### 3.2. Loss of independent ambulation

We identified 35 studies presenting evidence of prognostic indicators of disease progression in DMD measured in terms of loss of independent ambulation [11, 18–20, 38, 46, 51, 61–66, 68–91, 109, 126–130, 192, 193, 199]. In total, nine prognostic indicators were identified: age at diagnosis, age at onset of symptoms, ataluren treatment, DMD genetic modifiers, DMD mutation type, glucocorticoid exposure, eteplirsen treatment, height, and weight (Table 2). Prolonged independent ambulation was found in patients with later onset of symptoms [Level 2]; [83, 84] patients treated with glucocorticoids, including age at treatment initiation, duration of exposure, and pharmacological agent [Level 2]; [11, 18–20, 38, 46, 51, 61–64, 66, 70–82, 88, 199]; ataluren treatment [Level 2] [87, 109, 110, 192, 193]; eteplirsen treatment [Level 2] [126–130]; LTBP4 genotype [Level 2]; [65] lower limb surgery [Level 2] [89, 90] and mutations in exons 44 [Level 2] [11, 67, 73, 86, 88] and exons 3–7 [Level 2]; [11, 88] exon 8 [Level 4] [86, 88]; exon 45 [Level 4] [88, 199]; exon 53 [Level 4] [91];and the minor allele at rs1883832 [Level 4] [85]. Earlier loss of ambulation was found in patients with TG/GG genotype at the rs28357094 secreted phosphoprotein 1 (SPP1) promoter [Level 2]; [63–66] exon 51 skipping and exon 49–50 deletions [Level 4] [88]; and deletions in the dystrophin gene [Level 4] [61]. Older age at diagnosis (>4 years) has been shown to be a predictor of later loss of ambulation [Level 5] [74]. Finally, greater weight and lower height have been shown to predict delayed time to loss of ambulation in patients treated with glucocorticoids [Level 4] [68, 69].

### 3.3. Lower extremity and motor function

We found 47 studies presenting evidence of prognostic indicators of disease progression in DMD measured in terms of lower extremity and motor function [47, 51, 71, 75–77, 79–82, 87, 92, 96, 101, 119–131, 136, 138, 140, 141, 143–146, 150–152, 160–186, 188–198, 200–202]. In total, twelve prognostic indicators were identified: ataluren treatment, BMI, DMD genetic modifiers, DMD mutation type, drisapersen treatment, eteplirsen treatment, glucocorticoid exposure, height, TAS-205 treatment, vamorolone treatment, vitlolarsen treatment, and weight (Table 2). Glucocorticoid treatment, including dose, duration of exposure, and regimen, have been shown to be significantly associated with improvement in motor function as measured using the Scott functional score [Level 2] [140, 143], the Vignos scale [Level 4] [71, 96], muscle function measure [Level 4] [171, 172], improvements in the NorthStar Ambulatory Assessment (NSAA) scale [Level 1] [75–77, 131, 163–167, 170], the 6-minute walk test (6MWT) including duration of glucocorticoid exposure [Level 1] [131, 163–166, 175, 177–179], 10 Meter Walk/Run Test (10WRT) [Level 2] [79–81, 92, 96, 138, 174], 100 metre walk/run test [Level 3] [174], 9 metre walk/run test [Level 2] [47, 141, 144, 145], unspecified walking test [Level 4] [71], Supine-to-Stand (STS) test [Level 1] [47, 51, 71, 82, 92, 96, 101, 131, 136, 138, 141, 143, 145, 146, 160, 163–166], and 4-Stairs Climb Test (4SCT) including duration of exposure [Level 1] [47, 71, 82, 92, 96, 101, 131, 138, 141, 144, 145, 161, 163–166, 176]. Ataluren treatment has been shown to be significantly associated with better performance in timed function tests, including the 4SCT [Level 2] [87, 150, 189–193], the STS test [Level 3] [87, 192, 193], the 10WRT [Level 2] [150, 189–191], the NSAA [Level 2] [186–188], and the 6MWT [Level 2]; [150, 180–185, 189–191] treatment with TAS-205 has been shown to increase muscle volume index [Level 2] [196]; treatment with vitlolarsen associated with improved 10WRT, 6MWT, STS and NSAA [Level 2] [152]; treatment with vamorolone improves 6MWT [Level 3] [197] STS [197, 198], 10WRT [197, 198], 4SCT and NSAA [Level 4] [198]; treatment with drisapersen improves STS and 6MWT [Level 2] [168, 169, 200–202]. Eteplirsen treatment improves 6MWT [Level 2] [119–130, 162]. Greater height and weight have been shown to be significantly associated with decline in the 6MWT [Level 4]; [175] similarly, height, weight BMI and glucocorticoid exposure including duration are predictive of 4SC [Level 4] [161]. Finally, skip exon mutations has been shown to be significantly associated with 6MWT performance [Level 4] and [194, 195] Dp140 deletions associated with lower NSAA scores [Level 4] [151].

### 3.4. Muscle strength

We found 26 studies presenting evidence of prognostic indicators of disease progression in DMD measured in terms of muscle strength [18, 47, 71, 79, 80, 91, 92, 100, 101, 135–149, 153–159]. In total, five prognostic indicators were identified: DMD genetic modifiers, DMD mutation type, edasalonexent, glucocorticoid exposure and oxandrolone (Table 2). Specifically, glucocorticoid treatment, including dose, duration of exposure, and regimen, have been shown to be associated with muscle strength as quantified by the Medical Research Council (MRC) muscle power assessment scale [Level 2] [18, 71, 100, 135, 136, 138, 158], quantitative muscle testing (QMT) [Level 2] [92, 139], muscle mass as given by creatine excretion [Level 2] [137, 139, 142], manual muscle testing (MMT) [Level 2] [92, 139, 142, 145], myometric evaluation [Level 2] [140–146], unspecified muscle strength testing [Level 2] [101, 137], grip and pinch strength [Level 2] [47, 140, 141], Lovett’s test [Level 4]; [79, 80] and transverse relaxation time constant [Level 3] [159]. Edasalonexent improves the transverse relaxation time constant [Level 2] [153–157]. Oxandrolone improves muscle strength as given by MMT [Level 4] [149] and an unspecified measure [Level 2] [148]. Finally, GT/GG genotypes at the rs28357094 SPP1 promoter have been shown to be significantly associated with lower composite MRC scores and grip strength compared with the TT genotype [Level 4] [147]. and exon 53 deletions with lower pinch strength compared to all mutations not treatable by exon 53 skipping [Level 4] [91].

### 3.5. Respiratory health and function

We identified 35 studies presenting evidence of prognostic indicators of disease progression in DMD measured in terms of respiratory health and function [17–19, 21, 22, 28, 38, 47, 71, 77, 82, 92, 94–118, 132–134, 145, 199, 203]. In total, eight prognostic indicators were identified: ataluren treatment, DMD genetic modifiers, DMD mutation type, eteplirsen treatment, glucocorticoid exposure, idebenone treatment, ventilation support and weight (Table 2). Specifically, ataluren treatment has been shown to be significantly associated with improved forced vital capacity (FVC) [Level 2]; [103, 104, 109, 110] glucocorticoid treatment, including dose, duration of exposure, and regimen, with improved maximum inspiratory pressure (MIP) [Level 2] [92, 95, 96], maximum expiratory pressure (MEP) [Level 4] [94, 95], peak cough flow (PCF) [Level 4]; [94, 95] FVC [Level 2]; [17, 18, 21, 22, 38, 47, 71, 77, 82, 96–99, 101, 145] forced expiratory volume in 1 second (FEV_1_) [Level 2] [96, 107], maximum voluntary ventilation (MVV) [Level 2], [92, 101, 102], FVC [Level 4] [107], reduced need for ventilation [Level 4] [199] and peak expiratory flow rate (PEFR) [Level 3] [96, 98–100, 107] and pulmonary function preservation [Level 4] [19]. Duration of glucocorticoid exposure has also been linked to declining FVC levels [Level 4] [28]. Eteplirsen has been shown to be associated with an attenuation in respiratory function [Level 4] [108, 118] and reduced decline in FVC [Level 2] [113–117] and MEP [Level 2] [116, 117]; and idebenone reduces the decline in respiratory function as given by FVC [Level 2] [203], FEV1 [Level 2] [112] and PEF [Level 2] [111, 112, 133] as well as reducing bronchopulmonary adverse events [Level 2] [132]. Weight has been shown to be a significant predictor of need for full-time ventilation support [Level 4] [105]. Ventilation support has been shown to reduce the rate of decline of FVC [Level 4] [106]. Finally, Gly16 beta2-adrenergic receptor (ADRB2) polymorphism has been shown to be significantly associated with increased risk of requiring nocturnal ventilation support (compared with the Arg16 polymorphism) [Level 4] [105]; dystrophin protein 140 (Dp140)-related mutations with lower FVC [Level 4] [21, 22]; mutations in exon 44 with lower FVC, FEV1 and PEF [Level 4] [107]; skip 51 and 53 mutations with decreased FEV1, PEF and FVC [Level 4] [107]; splice site, skip 8 and skip 44 with increased FVC [Level 4] [107]; skip 8 and splice site mutations with increased FEV1 and increased PEF [Level 4] [107]; nonsense mutation with decreased FEV1 and FVC [Level 4] [107]; dominant G genotype at rs28357094 in the SPP1 promoter with reduced FVC and PEF [Level 4] [107]; additive T genotype at rs1883832 in the CD40 5’ untranslated region with reduced FVC, FEV1 and PEF [Level 4] [107];mutations in exon 8 with improved PEF [Level 4]; [21, 22]; cDMD deficit with worsened respiratory function [Level 3] [134]; and SPP1 and cluster of differentiation 40 (CD40) polymorphisms with reduced FVC and PEF, respectively [Level 4] [21, 22] with both mutations associated with NIV initiation [Level 4] [107].

### 3.6. Scoliosis

We identified 7 studies presenting evidence of prognostic indicators of disease progression in DMD measured in terms of risk of scoliosis [18, 46–50, 199]. In total, two prognostic indicators were identified: glucocorticoid exposure, and orthoses (Table 2). Specifically, glucocorticoid treatment, including duration of exposure, have been shown to significantly reduce the risk of developing scoliosis, including the degree of scoliosis and the need for spinal surgery [Level 3] [18, 46–50, 199]. Time in orthoses has been shown to be significantly related to scoliosis severity [Level 4] [50].

### 3.7. Survival

We identified 13 studies presenting evidence of prognostic indicators of disease progression in DMD measured in terms of survival [25, 42, 43, 49, 51–60]. In total, five prognostic indicators were identified: cardiac medication, glucocorticoid exposure, left ventricular assist devices, spinal surgery, and ventilation support (Table 2). Specifically, prolonged survival was found in patients treated with ACE inhibitors [Level 2] [42, 43] ACE inhibitors in combination with beta blockers, including timing of treatment initiation [Level 4]; [52] in patients treated with glucocorticoids (including duration of exposure) [Level 2]; [25, 49, 51] in patients receiving ventilation support [Level 4]; [53–59] and in those undergoing spinal surgery in combination with ventilation support [Level 4] [55].; and in those implanted with left ventricular assist devices in combination with cardiac medication [Level 4] [60].

### 3.8. Upper extremity function

We identified 5 studies presenting evidence of prognostic indicators of disease progression in DMD measured in terms of upper extremity function [51, 92, 93, 96, 140]. In total, two prognostic indicators were identified: glucocorticoid exposure (including pharmacological agent) and ATL1102 treatment (Table 2). Glucocorticoid treatment has been shown to significantly retain hand-to-mouth function as measured using the Brooke score [Level 2]; [51, 92, 96, 140] and deflazacort (DFZ) exposure significantly delays loss of hand-to-mouth function compared to prednisone (PDN) [Level 2] [51]. Treatment with ATL1102 improves upper limb function in non-ambulant boys as given by performance of upper limb (PUL) scores [Level 2] [93].

## 4. Discussion

In many disease areas, including DMD, RCTs are commonly unavailable, resulting in the need to indirectly compare treatment effects, for example, by pooling individual patient-level data from multiple sources. However, to derive reliable estimates, it is necessary to ensure that the samples considered are comparable with respect to factors significantly affecting the clinical progression of the disease. To help inform such analyses, the objective of this study was to review and synthesise the published evidence of prognostic indicators of disease progression in DMD. From our literature search, we identified 23 factors significantly affecting disease progression outcomes in DMD, namely age at diagnosis, age at onset of symptoms, ataluren treatment, ATL1102, BMI, cardiac medication, DMD genetic modifiers, DMD mutation type, drisapersen, edasalonexent, eteplirsen, glucocorticoid exposure, height, idebenone, lower limb surgery, orthoses, oxandrolone, spinal surgery, TAS-205, vamorolone, vitlolarsen, ventilation support, and weight. Of these, two endogenous and two exogenous core prognostic indicators were designated, each supported by a high level of clinical evidence.

The most commonly examined prognostic indicator identified in the literature related to treatment with glucocorticoids–the cornerstone of the current pharmacological management of DMD. This core exogenous factor was found to significantly impact a wide range of disease progression outcomes, including loss of independent ambulation, lower extremity and motor function, muscle strength, respiratory health and function, survival, and upper extremity function (high level of evidence); cardiac health and function (moderate level of evidence); and possibly risk of developing scoliosis (low level of evidence). The body of evidence, spanning a total of 73 individual studies, encompassed various commonly reported features of glucocorticoid therapy, such as age at treatment initiation, dose, duration of exposure, pharmacological agent, and regimen.

The second exogenous core prognostic indicator of disease progression in DMD was cardiac medication, supported by data from a total of 13 studies of varying levels of evidence (Fig 2). As expected, this indicator only concerned cardiac health and function (with the exception of a single study of low evidence level showing an impact on survival). Even so, bearing in mind that cardiomyopathy has emerged as one of the leading causes of death in the aging DMD population in the presence of the routine use of mechanical ventilation support [12], the significance of this indicator should not be underestimated, in particular when comparing samples encompassing patients residing in more advanced stages of the disease.

The two endogenous core prognostic indicators of disease progression in DMD identified in our review were DMD genetic modifiers and DMD mutation type. Although more research is needed to quantify the impact of specific modifiers and mutations, emerging data show that these genetic aspects may play a non-trivial role in the overall progression of the disease. These findings underscore the importance of collecting genetic data from DMD patients as part of studies and patient registries.

Our study is subject to three specific limitations. First, our review did not cover grey literature, which means that evidence for some indicators of disease progression in DMD might have not been fully identified. However, given the comprehensive scope of our search and the limited body of clinical evidence disseminated in non-indexed journals, the impact of this limitation is expected to be negligible (in particular in terms of detecting novel prognostic indicators currently not included in our synthesis). Second, for interpretation of results, it is important to keep in mind that our study did not seek to assess the efficacy or effectiveness of current disease interventions, nor the sensitivity of specific indicators, but rather identify factors that have been shown to significantly alter the clinical progression of DMD (irrespective of magnitude). Although we only considered statistically significant factors, this means that it is not possible to discern the relative clinical importance, or relevance, of included indicators. Finally, the fact that we only reported statistically significant and not also non-significant results means that we were more likely to accept false positive than false negative conclusions of specific indicators. That being said, collating and synthesizing also non-significant results, of which a non-trivial proportion (β) would be expected to be false, were outside the scope of this review.

In conclusion, we identified a total of 23 prognostic indicators of disease progression in DMD, of which cardiac medication, DMD genetic modifiers, DMD mutation type, and glucocorticoid exposure were designated core indicators significantly affecting a wide range of clinical outcomes. Our up-to-date summary of prognostic indicators in DMD should be helpful to inform the design of comparative analyses and future data collection initiatives in this patient population.

## Supporting information

S1 Checklist PRISMA 2009 checklist. (PDF)

S1 Appendix Search strings. (DOCX)
