# Correction: Prognostic indicators of disease progression in Duchenne muscular dystrophy: A literature review and evidence synthesis

**DOI:** 10.1371/journal.pone.0315682

**Published:** 2024-12-09

**Authors:** Nermina Ferizovic, Jessica Summers, Igor Beitia Ortiz de Zárate, Christian Werner, Joel Jiang, Erik Landfeldt, Katharina Buesch

The third author’s initials appear incorrectly in the citation. The correct citation is: Ferizovic N, Summers J, Beitia Ortiz de Zárate I, Werner C, Jiang J, Landfeldt E, et al. (2022) Prognostic indicators of disease progression in Duchenne muscular dystrophy: A literature review and evidence synthesis. PLoS ONE 17(3): e0265879. https://doi.org/10.1371/journal.pone.0265879.
