# Supplementary material for: Prognostic indicators of disease progression in Duchenne muscular dystrophy: A literature review and evidence synthesis
Source: PLoS One. 2022 Mar 25;17(3):e0265879. doi: 10.1371/journal.pone.0265879 (PMC8956179; doi:10.1371/journal.pone.0265879)
Supplement: S1 Appendix — (DOCX) [file pone.0265879.s002.docx]

S1 Appendix

**eTable 1: Search terms for MEDLINE ALL (including MEDLINE daily, MEDLINE ePub ahead of print, MEDLINE (R) In-Process), via Ovid (Search 1: 1946 to January 23, 2020, Search 2: 1946 to April 23, 2021)**

| **Number** | **Search terms** | **Hits 24/01/2020** | **Hits 26/04/2021** |
| --- | --- | --- | --- |
| 1 | exp Muscular Dystrophy, Duchenne/ | 5242 | 5786 |
| 2 | (Duchenne and dystro*).mp. | 11758 | 12576 |
| 3 | 1 or 2 | 11758 | 12576 |
| 4 | (prognos* or (disease adj3 course) or (disease adj3 impact) or natural history or (disease adj3 predict*) or (disease adj3 outcome) or (disease adj3 progres*)).mp. | 1159013 | 1280238 |
| 5 | 3 and 4 | 1294 | 1449 |
| 6 | (comment or letter or editorial or notes or review).pt. | 4374014 | 4707299 |
| 7 | (exp animals/ or exp invertebrate/ or animal experiment/ or animal model/) and (human/) | 18256130 | 19185635 |
| 8 | (exp animals/ or exp invertebrate/ or animal experiment/ or animal model/) not 7 | 4666981 | 4816438 |
| 9 | 6 or 8 | 8827341 | 9301933 |
| 10 | 5 not 9 | 857 | 955 |
| 11 | Limit 10 to dt=20200123-20210426 | - | 117 |

**eTable 2: Search terms for Embase, via Ovid (Search 1: 1974 to 2020 January 23, Search 2: 1974 to April 23 2021)**

| **Number** | **Search terms** | **Hits 24/01/2020** | **Hits 26/04/2021** |
| --- | --- | --- | --- |
| 1 | exp Duchenne muscular dystrophy/ | 15685 | 17024 |
| 2 | (Duchenne and dystro*).mp. | 18983 | 20475 |
| 3 | 1 or 2 | 18983 | 20475 |
| 4 | (prognos* or (disease adj3 course) or (disease adj3 impact) or natural history or (disease adj3 predict*) or (disease adj3 outcome) or (disease adj3 progres*)).mp. | 1758661 | 1931528 |
| 5 | 3 and 4 | 2529 | 2814 |
| 6 | (comment or letter or editorial or notes or review).pt. | 4274669 | 4651024 |
| 7 | (exp animal/ or exp invertebrate/ or animal experiment/ or animal model/) and (human/) | 20334261 | 22307587 |
| 8 | (exp animal/ or exp invertebrate/ or animal experiment/ or animal model/) not 7 | 5044619 | 5294373 |
| 9 | 6 or 8 | 9159735 | 9776724 |
| 10 | 5 not 9 | 1712 | 1911 |
| 11 | Limit 10 to dc=20200123-20210426 | - | 194 |

**eTable 3: The Cochrane Database of Systematic Reviews search strategy (via Cochrane Library interface) and The Cochrane Central Register of Controlled Trials**

| **Number** | **Search terms** | **Hits 24/01/2020** | **Hits 26/04/2021** |
| --- | --- | --- | --- |
| 1 | [mh "Muscular Dystrophy, Duchenne"] | 192 | 228 |
| 2 | (Duchenne and dystro*):ti,ab,kw | 704 | 753 |
| 3 | #1 or #2 | 704 | 753 |
| 4 | (prognos* or (disease NEAR/3 course) or (disease NEAR/3 impact) or natural history or (disease NEAR/3 predict*) or (disease NEAR/3 outcome) or (disease NEAR/3 progres*)):ti,ab,kw | 90594 | 95110 |
| 6 | #3 and #4 | 130 | 143 |
| 7 | #5 with Publication Year from 2020 to 2021, with Cochrane Library publication date from Jan 2020 to May 2021, in Trials | - | 8 |

Cochrane Central Register of Controlled Trials Issue 1 of 12, January 2020 (n=128), Cochrane Database of Systematic Reviews Issue 1 of 12, January 2020 (n=2)

Cochrane Central Register of Controlled Trials Issue 3 of 12, March 2021 (n=8), Cochrane Database of Systematic Reviews Issue 3 of 12, March 2021 (n=0)
